# Acute effects of prismatic adaptation on penalty kick accuracy and postural control in young soccer players: A pilot study

**DOI:** 10.1016/j.heliyon.2024.e30515

**Published:** 2024-04-30

**Authors:** Valerio Giustino, Rosario Emanuele Bonaventura, Giuseppe Messina, Antonino Patti, Guglielmo Pillitteri, Simona Pajaujiene, Antonio Paoli, Antonio Palma, Antonino Bianco, Massimiliano Oliveri, Giuseppe Battaglia

**Affiliations:** a Sport and Exercise Sciences Research Unit, Department of Psychology, Educational Science and Human Movement, University of Palermo, Palermo, Italy; b Department of Psychology, Educational Science and Human Movement, University of Palermo, Palermo, Italy; c Department of Human Sciences and Promotion of the Quality of Life, San Raffaele University, Rome, Italy; d PLab Research Institute, Palermo, Italy; e Department of Coaching Science, Lithuanian Sports University, Kaunas, Lithuania; f Department of Biomedical Sciences, University of Padova, Padova, Italy; g Regional Sports School of Italian National Olympic Committee (CONI) Sicilia, Palermo, Italy; h Department of Biomedicine, Neuroscience and Advanced Diagnostics, University of Palermo, Palermo, Italy

**Keywords:** Sport neuroscience, Sport performance, Prismatic adaptation, Visuomotor performance, Visuomotor procedure, Visual training, Kinematic, Body posture

## Abstract

**Background:**

Prismatic adaptation (PA) is a visuomotor technique using prismatic glasses that are capable of moving the visual field and to affect the excitability of certain brain areas. The aim of this pilot study was to explore potential acute effects of PA on penalty kick accuracy and postural control in youth soccer players.

**Methods:**

In this randomized crossover study, seven young male soccer players performed three PA sessions (rightward PA, r-PA; leftward PA, l-PA; sham PA, s-PA) with a washout period of 1-week between them. Immediately before and after each PA session, penalty kick accuracy and postural control were assessed.

**Results:**

We detected an increase in penalty kick accuracy following PA, regardless of the deviation side of the prismatic glasses (F_1,5_ = 52.15; p = 0.08; ηp^2^ = 0.981). In detail, our results showed an increase in the penalty kick accuracy toward the right target of the football goal following r-PA and toward the left target of the football goal following l-PA. We detected a significant effect on the sway path length (F_2,12_ = 10.42; p = 0.002; ηp^2^ = 0.635) and the sway average speed (F_2,12_ = 9.17; p = 0.004; ηp^2^ = 0.605) parameters in the stabilometric test with open eyes following PA, regardless of the deviation side of the prismatic glasses. In detail, our results showed a significant difference in both the stabilometric parameters (p = 0.016 and p = 0.009, respectively) only following l-PA.

**Conclusion:**

The findings of this pilot study indicate that PA could positively affect penalty kick accuracy and postural control suggesting that PA could be used as a visual training technique in athletes.

## 1 Introduction

The athlete's sport performance depends on several domains including physical, technical/tactical skills, and other abilities such as postural control and visuomotor performance [[1], [2], [3], [4], [5], [6], [7], [8], [9], [10], [11], [12], [13], [14]]. Over time, different devices and procedures have been developed with the aim of improving these abilities [[1], [2], [3], [4], [5], [6], [7], [8], [9], [10], [11], [12], [13], [14]].

As one of the primary perceptual-cognitive skills for environment perception, the visuomotor performance has an essential function in sport performance [10]. In fact, the literature shows several studies that have investigated the influence of visual training on performance of different sports such as badminton, volleyball, and soccer [[7], [8], [9], [10], [11], [12], [13]]. For example, a visual procedure that uses stroboscopic glasses capable of determining intermittent vision has been used in athletes with the aim of improving sport performance [[7], [8], [9], [10], [11], [12], [13]].

As for soccer, visual skills such as peripheral vision, depth perception, visual reaction time, and visual tracking of a moving object are of fundamental importance for players [13,[15], [16], [17], [18]]. For these reasons, many articles have demonstrated the role of visual training in this sport [13,[15], [16], [17], [18]]. For example, Wilkins et al. studied the influence of a visual training program using stroboscopic glasses on visual-perceptual skills in young soccer goalkeepers [13]. Although the authors found no differences in almost all measurements investigated, goalkeepers who had worn stroboscopic glasses reported enhancements in visual response time compared to those who did not use them [13]. In the study by Theofilou et al., a group of young players who had performed the usual soccer training program integrated with a visual training program showed significant improvements in simple reaction time, repeated sprints, and visuomotor function [17]. However, in most of the measurements carried out, no significant differences were found compared to a group of young players who had only performed the regular soccer training program [17]. Nimmerichter et al. explored the effects of a video-based visual training in young soccer players detecting significant improvements in decisions response time, successful decisions, and reactive agility sprint time [18].

These studies emphasize the use of visuomotor procedures in sports and, based on these premises, performance enhancements in athletes could also be provided by prismatic adaptation (PA). PA is a visuomotor technique using prismatic glasses that are capable of moving the visual field to the right (rightward deviation) or to the left (leftward deviation) according to the deviation side of the prismatic glasses (left-based or right-based, respectively). Previous studies have shown that the visual-motor system is able to adapt to the lateral shift of the visual field induced by prismatic glasses [[19], [20], [21]]. For this reason, this visuomotor technique has been investigated in people who struggle to explore a visual hemifield [19,[22], [23], [24]]. More recently, PA has been studied in healthy people with the aim of exploring the brain areas activated by this visuomotor technique [[25], [26], [27]]. The findings demonstrated that PA is capable to affect the excitability of the primary motor cortex, parietal cortex, and cerebellum, showing that this visuomotor technique could affect the functions related to these brain regions [21,[28], [29], [30]]. In fact, the research has studied potential effects of this visuomotor technique on skills such as walking, aiming, throwing, and postural control [29,[31], [32], [33], [34]]. However, PA has not yet been investigated on sport performance.

In soccer, visuospatial and spatiotemporal skills as well as postural control are of crucial importance for performance, especially in specific skills such as the penalty kick [35,36]. We hypothesized that, based on the known effects of PA, this visuomotor technique could positively influence sport performance in soccer players.

This pilot study aimed to explore potential acute effects of PA on penalty kick accuracy and postural control in youth soccer players. The novelty is that no previous research, to date, has studied this visuomotor procedure in athletes.

## 2 Materials and methods

### 2.1 Study design

A randomized crossover design was adopted for this study. All participants performed three PA sessions (rightward PA, r-PA; leftward PA, l-PA; sham PA, s-PA) with a 1-week washout period between them. The three PA sessions were randomized via a simple randomization process in which participants were randomly assigned to treatment groups (i.e., the PA sessions) using Graphpad Prism software (GraphPad Software Inc., San Diego, CA, USA). Immediately before and after each PA session, penalty kick accuracy and postural control were assessed.

### 2.2 Participants

Seven young male soccer players (age: 15.86 ± 0.38 years; height: 173.57 ± 6 cm; weight: 65.86 ± 8.91 kg), belonging to a Sicilian team (Italy) of the “Under 17 Regional Championship”, were recruited. To be included in the study all participants had to meet the following inclusion criteria: (1) at least 5 years of playing experience; (2) no injury at the time of the enrolment in the study; (3) no injuries in the 6 months prior to the enrolment in the study; (4) any playing position as long as they were the best penalty takers on the team; (5) right-handedness and right-footedness. The exclusion criteria were as follows: (1) goalkeeper as playing position; (2) left-handedness and right-footedness.

For study participation, the parents of all participants signed a written informed consent. The study was approved by the Bioethics Committee of the University of Palermo (n. 95/2022) in accordance with the criteria of the Declaration of Helsinki.

### 2.3 Experimental protocol

#### 2.3.1 Prismatic adaptation (PA)

Each participant performed three sessions of PA, each with a different deviation side of the prismatic glasses (i.e., rightward PA, r-PA; leftward PA, l-PA; sham PA, s-PA) with a 1-week washout period between the sessions.

**Fig. 1:**
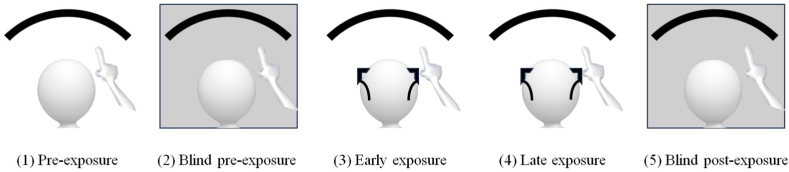
PA procedure consisted of pointing movements. Legend. Phase 1, pre-exposure (no prisms); phase 2, blind pre-exposure (no prisms); phase 3, early exposure (wearing prisms); phase 4, late exposure (i.e., adaptation - wearing prisms); phase 5, blind post-exposure (i.e., after-effect - no prisms).

The PA procedure used was identical to that of our previous work and consisted of 5 phases including 180 pointing movements of which 30 pointing movements for each of the phases 1, 2, and 5, and 90 pointing movements for both the phases 3 and 4 (Fig. 1) [37]. During each session of PA each participant was asked to carry out pointing movements with the index finger of the right hand towards one of the three vertical lines on a curved Plexiglas panel pseudorandomly indicated by the same experimenter (R.E.B.). The curved Plexiglas panel covered a visual angle of 120° and was graduated with parallel vertical lines each corresponding to one degree of the visual angle. The three vertical lines pseudorandomly indicated by the same experimenter (R.E.B.) corresponding to the central position (0°), left position (21° to the left), and right position (21° to the right). The same experimenter (R.E.B.) measured the pointing accuracy by noting the pointing errors from the vertical line indicated (+1 or −1 for each degree of the visual angle to the right or to the left, respectively).

#### 2.3.2 Penalty kick accuracy

Penalty kick accuracy was assessed immediately prior and following each session of PA through a series of 30 penalty kicks without goalkeeper, aimed towards one of three targets of a football goal and pseudorandomly indicated by the same experimenter (V.G.) through verbal commands. Participants were explained that the experimenter's verbal commands were “right” or “left” to indicate that the ball should be kicked into the goal as close as possible to the right post and as close as possible to the left post, respectively. The verbal command “center” indicated that the ball should be kicked towards the center of the goal. The same experimenter (V.G.) placed the ball on the spot for each penalty kick and gave the verbal command regarding the towards in which the ball should be kicked. A 30-s rest was given among penalty kicks.

Each participant kicked with the dominant foot (i.e., right foot) all the penalty kicks with a self-selected length run-up and angle run-up. To determine their dominant foot, players were asked which foot they preferred to control, pass, and throw the ball with [38,39].

Prior each series of penalty kicks, the participants performed a self-selected warm-up, lasting approximately 15 min, and three penalty kicks as familiarization (one penalty kick for each target goal, i.e., right, center, left indicated through the experimenter's verbal commands). Since it is known that warm-up could affect performance [40,41], the self-selected warm-up was their usual warm-up and was the same for all participants. In fact, all participants belonged to the same team and their coach, who was present throughout the experimental protocol, conducted the warm-up which included light jogging followed by dynamic lower extremity movements and joint mobility exercises.

**Fig. 2:**
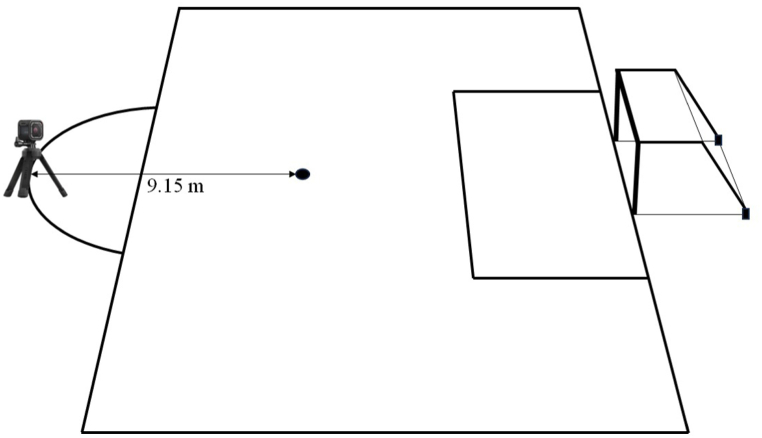
Setting for recording penalty kick sequences. Legend. The distance from the action camera (positioned in the center of the front edge of the penalty area) and the penalty spot was 9.15 m.

During the experimental protocol, an action camera (GoPro HERO8, GoPro Inc., San Mateo, CA, US) was positioned in the center of the front edge of the penalty area (i.e., 9.15 m from the penalty spot) in order to frame the goal. Each series of penalty kicks was recorded through the action camera. Fig. 2 shows the setting for recording each series of penalty kicks.

Subsequently, a video analysis was carried out by the same experimenter (V.G.) using a software designed for sport analysis (Kinovea®, version 0.9.4) that made it possible to assess the accuracy of each penalty kick by measuring the distance from the target where the penalty was taken to the actual distance from this. Each series of penalty kicks was recorded in one video. The videos were analysed in slow motion and, for each penalty kick, the freeze frame of the exact moment the ball entered the football goal was used to measure the distance from the target to the effective point where the ball arrived. The zoom function was used in the freeze frame to increase accuracy measurement. In detail, accuracy was measured as the error in horizontal dimension as follows: a) from the right edge of the ball to the inside edge of the right post for penalty kicks kicked on the right; b) from the left edge of the ball to the inside edge of the left post for penalty kicks kicked on the left; c) from the center of the ball to the center of the target for penalty kicks kicked on the center.

#### 2.3.3 Postural control

Immediately prior and following each session of PA, a stabilometric test was performed in order to assess any differences on postural control.

Using the Freemed™ platform and the Freestep™ software (Sensor Medica®, Guidonia Montecelio, Roma, Italia), the stabilometric test was conducted in two distinct modes: with open eyes (OE) and with closed eyes (CE) using a sampling frequency of 50 Hz. The validity and the reliability of the device have been previously studied [42,43].

Participants were asked to maintain their normal upright posture for 51.2 s, barefoot, and with feet placed side-by-side forming an angle of 30° and both heels 2 cm apart. The characteristics and settings adopted for the stabilometric test were in accordance with the standards for stabilometric assessment [[44], [45], [46]].

For statistical analysis, we considered the following parameters of the center of pressure (CoP): sway path length (SPL); sway ellipse area (SEA); CoP components in the medio-lateral (ΔX; X-mean) and anterior-posterior (ΔY; Y-mean) directions, sway average speed.

## 3 Statistical analysis

### 3.1 Participants

The minimum sample size required to reach a power of 80 % (f = 0.40, α = 0.05) was calculated a priori, assuming an ANOVA design, using G*Power software (v. 3.1.9.2; Heinrich Heine University, Düsseldorf, Germany). Moreover, a post hoc analysis was computed to detect the achieved sample size power.

### 3.2 Penalty kick accuracy

Penalty kick accuracy was analysed trough a 3 × 2 ANOVA with deviation (l-PA vs r-PA vs s-PA) as between subjects factor and time (pre-prism exposure vs post-prism exposure) as within subjects factor. Moreover, a 3 × 3 × 2 ANOVA was performed to deeply investigate penalty kick accuracy effects with deviation (l-PA vs r-PA vs s-PA) as between subjects factor, target position of the football goal (left vs center vs right) and time (pre-prism exposure vs post-prism exposure) as within subjects factors.

The partial eta-squared was carried out to measure the effect size.

The Bonferroni post-hoc tests were computed to examine the factors main effects and the interaction effects when appropriate.

### 3.3 Postural control

For both OE and CE conditions of the postural control measurement, the parameters were investigated trough a 3 × 2 ANOVA with deviation (l-PA vs r-PA vs s-PA) as between subjects factor and time (pre-prism exposure vs post-prism exposure) as within subjects factor.

The partial eta-squared was carried out to measure the effect size.

The Bonferroni post-hoc tests were computed to examine the factors main effects and the interaction effects when appropriate.

We set α < 0.05 as the significance level. All statistical analyses were conducted using IBM SPSS Statistics software 26 (International Business Machines Corporation, Armonk, New York, United States).

## 4 Results

### 4.1 Participants

The minimum sample size required a priori was of 12 participants. The post hoc power analysis showed that with a sample size of 7 participants we achieved a power of 52 %.

### 4.2 Penalty kick accuracy

**Fig. 3:**
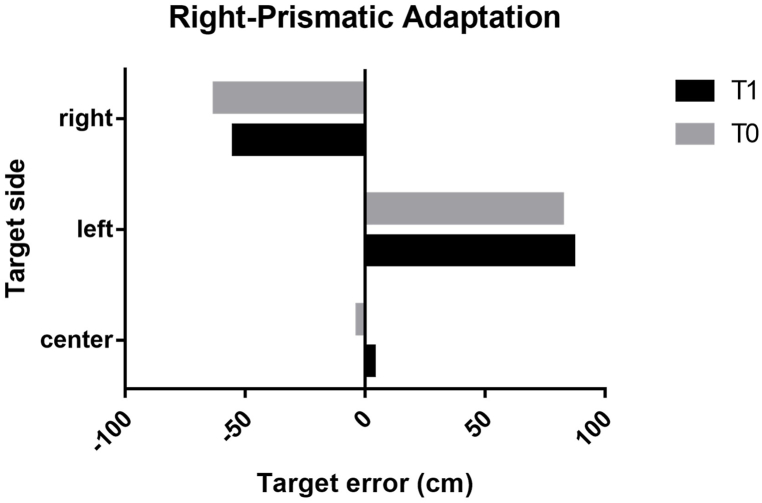
Accuracy of the penalty kick after PA with right deviation. Legend. T0, pre-prismatic adaptation; T1, post-prismatic adaptation; right, right target of the football goal; left, left target of the football goal; center, center target of the football goal.

**Fig. 4:**
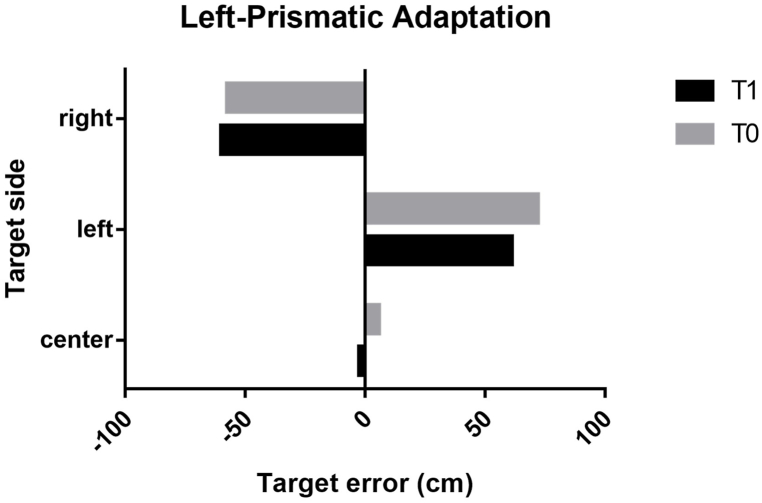
Accuracy of the penalty kick after PA with left deviation. Legend. T0, pre-prismatic adaptation; T1, post-prismatic adaptation; right, right target of the football goal; left, left target of the football goal; center, center target of the football goal.

We detected an increase in penalty kick accuracy following PA, regardless of the deviation side of the prismatic glasses (F_1,5_ = 52.15; p = 0.08; ηp^2^ = 0.981). In detail, our results showed an increase in the penalty kick accuracy toward the right target of the football goal following r-PA (Fig. 3) and toward the left target of the football goal following l-PA (Fig. 4). No significant interaction was detected between the deviation side of the prismatic glasses, prior and following PA, and penalty kick accuracy toward the target of the football goal.

### 4.3 Postural control

**Fig. 5:**
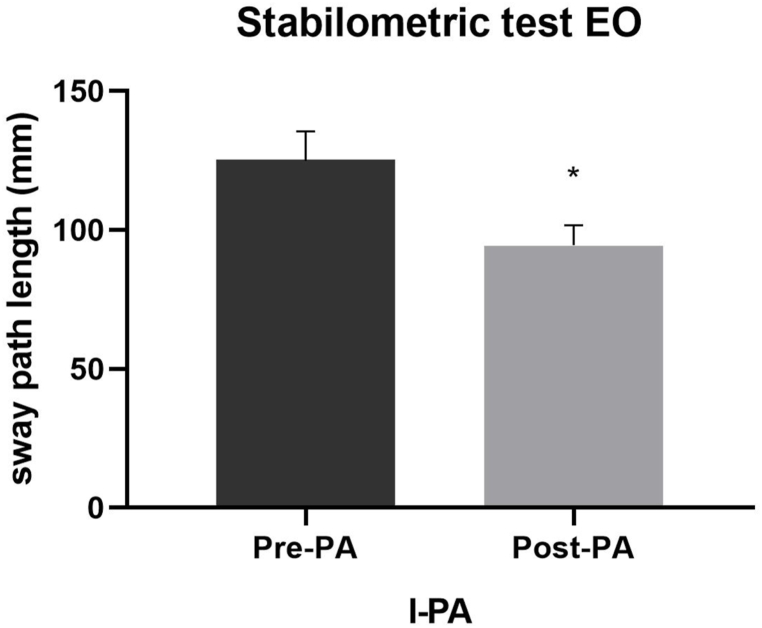
Post-hoc analysis of the sway path length parameter after PA with left deviation. Legend. l-PA, left-prismatic adaptation; Pre-PA, pre-prismatic adaptation; Post-PA, post-prismatic adaptation.

**Fig. 6:**
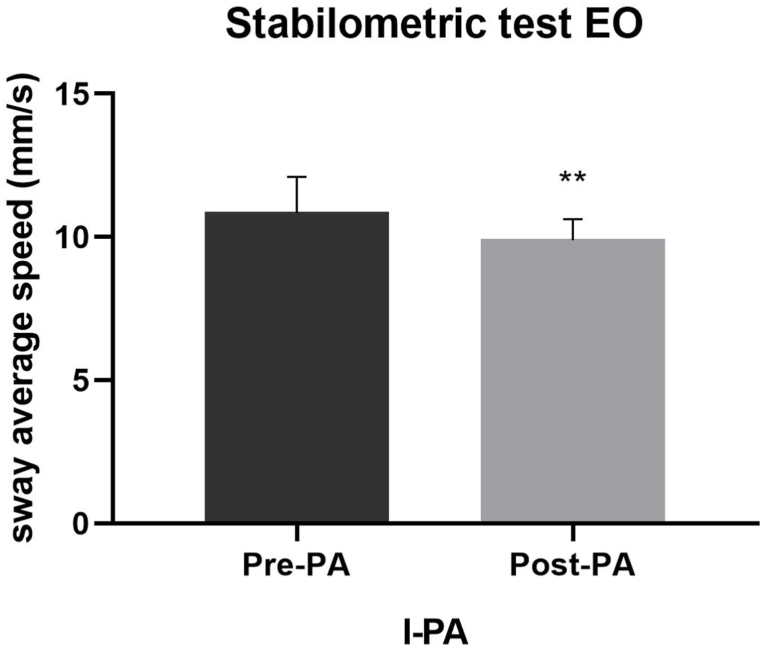
Post-hoc analysis of the sway average speed parameter after PA with left deviation. Legend. l-PA, left-prismatic adaptation; Pre-PA, pre-prismatic adaptation; Post-PA, post-prismatic adaptation.

We detected a significant effect on the sway path length (F_2,12_ = 10.42; p = 0.002; η_p_^2^ = 0.635) and the sway average speed (F_2,12_ = 9.17; p = 0.004; η_p_^2^ = 0.605) parameters in the stabilometric test with OE following PA, regardless of the deviation side of the prismatic glasses. In detail, the post-hoc analysis revealed a significant difference in both the sway path length (125.37 vs 94.35; p = 0.016) (Fig. 5) and the sway average speed (10.88 vs 9.95; p = 0.009) (Fig. 6) parameters only following l-PA.

## 5 Discussion

This pilot study aimed to explore potential acute effects of PA on penalty kick accuracy and postural control in youth soccer players.

Previous studies have demonstrated that PA can modulate the excitability of the primary motor cortex, parietal cortex, and cerebellum influencing their related functions such as visuospatial and spatiotemporal skills as well as postural control [21,[28], [29], [30]]. In soccer, visual skills such as peripheral vision, depth perception, visual reaction time, and visual tracking of a moving object are of fundamental importance for players [13,[15], [16], [17], [18]]. In fact, many research groups have demonstrated the influence of visual training on sport performance in soccer players [13,[15], [16], [17], [18]].

These findings indicate that PA could positively affect penalty kick accuracy and postural control in soccer players. To some extent our results support our hypothesis on the employment of PA to improve sport performance in athletes.

In soccer, accuracy is a fundamental element and represents the ability to kick the ball in an exact area [47]. The objective of improving penalty kick accuracy is one of the widely studied topics in soccer. Over time, various techniques and strategies have been used to investigate any improvements in penalty kick performance [36,[48], [49], [50]]. For example, Navarro et al. studied the differences of implicit and explicit training methodologies on penalty kick [36]. Authors found that while both training methodologies showed comparable levels of decision-making, implicit training led to improved penalty kick accuracy [36]. Scurr and Hall investigated the differences of approach angle (i.e., 30°, 45°, and 60°) on penalty kick accuracy and three-dimensional kinematics in right-dominant foot soccer players finding no significant differences in kicking accuracy and ball velocity, while kinematics differences were found among the approach angles [48]. Van den Tillaar and Fuglstad examined the relationship between speed and accuracy in penalty kick in experienced soccer players by using commands that prioritized speed, accuracy, or both [49]. Among the main findings, the authors found that when accuracy was the primary goal, accuracy increased and speed decreased. Furthermore, penalty kick accuracy was higher when kicking to the contralateral side [49]. Makaruk et al. investigating the key factors that directly affected penalty kick accuracy, reported that a mix of attentional and motivational factors may improve penalty kick performance [50].

In the current study, we did not require participants for a specific type of kick (inside-of-the-foot kick, instep kick, outside-of-the-foot kick), or a specific run-up length, or a specific angle of approach, but kicking as accurately as possible regardless of ball speed [51]. We are aware that accuracy and speed are two characteristics that can be required at the same time for the success of a penalty kick [52]. For this reason, we believe that both characteristics should be studied carefully and separately. In fact, a complete and selective knowledge of the effects that a training technique, such as PA, can led to each single movement characteristic is more suitable for knowing the specific effects on performance [53,54]. In this way, we suggest that for the success of a penalty kick it would be appropriate to investigate the effects of PA first on accuracy and then on speed, to finally integrate the two variables and studying the global effect of this technique. By the same principle the use of PA in a training program should first be selective for accuracy and then for speed and subsequently it should integrate both characteristics.

As for the type of kick, a seminal study showed that, regardless of the type of kick (in particular, between inside-of-the-foot kick and instep kick), the thigh, shank, and foot form a three-link kinetic chain for the kicking leg [55]. Based on this premise and to the best of our knowledge, previous studies which analysed any differences between the types of kick found differences only in speed and, among others, no significant difference in the angular and linear kinematic parameters of the sagittal plane [51,56]. Since no findings were reported in accuracy, this knowledge allowed us to avoid any bias regarding the different type of kick adopted by the participants.

Similarly, concerning the run-up, Scurr and Hall did not detect difference in accuracy between different approach angles [48].

Moreover, the participants recruited were right-footedness and were asked to kick with the dominant foot. This approach was adopted because it is well known that exist differences in motor control between preferred and non-preferred leg kicks in soccer players [57].

Furthermore, the choice to develop the sequence of penalty kicks without the presence of the goalkeeper was made to involve the keeper-independent strategy in which participants can kick toward a target by ignoring the goalkeeper's actions that has been suggested as the preferred strategy for kicking a penalty kick [58,59]. This was done to avoid the presence of a confounding variable. In fact, it was demonstrated that the presence of a goalkeeper impairs the accuracy of a penalty kick [58].

The majority of studies in the field of penalty kicks has been on spatial decision making, that is the goal side where kicked, and in these terms accuracy plays a crucial role [60]. We measured penalty kick accuracy as the error in horizontal dimension using an advanced method, i.e., through video analysis as similarly carried out by a previous work [52] and based on a similar approach of a seminal method developed by Finnoff et al. [47]. This allowed as to measure the distance from the target to the effective point where the ball entered to the football goal.

The results concerning both the improvement in penalty kick accuracy and the significant effect on postural control after PA confirmed the effectiveness of this procedure in modulating the activity of different brain regions. In particular, the improvement in penalty kick accuracy could be related to the activation of the parietal cortex and the primary motor cortex that occurs with this procedure and which consequently influence the related functions such as the visuospatial attention, orienting of attention, spatial/temporal representation, and quality of visually guided actions [21,28,29], all features fundamental to take a penalty kick. In line with our results, previous studies have reported beneficial effects of PA on throwing accuracy [[61], [62], [63]]. In this pilot study we translated the study of PA on throwing accuracy of upper limb to kicking accuracy of lower limb.

According to previous research, this technique has an effect on the activity of the cerebellum [30] and this activation can have an influence on postural control, as we detected in this study. Indeed, our findings support the existing literature reporting that PA positively affect postural control [64].

Among the limitations of the study, we reported: (1) the small sample size considered to detect the effects of this visuomotor procedure; (2) and the use of this visuomotor procedure in only a sport-specific skill, i.e., penalty kick, and in only a physical characteristic, i.e., postural control. Another limitation could be the use of a software for video analysis of the penalty kick which could be operator-dependent.

Based on the acute effects found, coaches could use PA as a visual training technique for improvements on visuomotor performance in athletes.

## 6 Conclusion

The findings of this pilot study indicate that PA could positively affect penalty kick accuracy and postural control in youth soccer players. These results suggest that the use of this visuomotor procedure could be applied, as well as other visual training techniques, to athletes. Nevertheless, these results should be interpreted with caution and further research is needed to better understand the effectiveness of PA on sport performance in athletes practicing different sports.

## Data availability statement

The data of this study are available from the corresponding author upon reasonable request.

## CRediT authorship contribution statement

**Valerio Giustino:** Writing – original draft, Validation, Methodology, Formal analysis, Data curation, Conceptualization. **Rosario Emanuele Bonaventura:** Writing – original draft, Validation, Methodology, Formal analysis, Data curation, Conceptualization. **Giuseppe Messina:** Validation, Project administration, Conceptualization. **Antonino Patti:** Writing – review & editing, Visualization, Formal analysis. **Guglielmo Pillitteri:** Visualization, Validation. **Simona Pajaujiene:** Visualization, Validation. **Antonio Paoli:** Visualization, Validation. **Antonio Palma:** Visualization, Validation, Supervision. **Antonino Bianco:** Visualization, Validation, Supervision. **Massimiliano Oliveri:** Writing – review & editing, Supervision, Project administration, Methodology, Conceptualization. **Giuseppe Battaglia:** Writing – review & editing, Supervision, Project administration, Methodology, Conceptualization.

## Declaration of competing interest

The authors declare that they have no known competing financial interests or personal relationships that could have appeared to influence the work reported in this paper.
